# Optimizing rice plant photosynthate allocation reduces N_2_O emissions from paddy fields

**DOI:** 10.1038/srep29333

**Published:** 2016-07-05

**Authors:** Yu Jiang, Xiaomin Huang, Xin Zhang, Xingyue Zhang, Yi Zhang, Chengyan Zheng, Aixing Deng, Jun Zhang, Lianhai Wu, Shuijin Hu, Weijian Zhang

**Affiliations:** 1 Institute of Applied Ecology, Nanjing Agricultural University, Nanjing 210095, China; 2 Department of Plant Pathology, North Carolina State University, Raleigh, NC 27695, USA; 3 Institute of Crop Sciences, Chinese Academy of Agricultural Sciences / Key Laboratory of Crop Physiology and Ecology, Ministry of Agriculture, Beijing 100081, China; 4 The High School Affiliated to Renmin University of China, Beijing 100080, China; 5 Sustainable Soils and Grassland Systems Department, Rothamsted Research, North Wyke, Okehampton EX20 2SB, UK

## Abstract

Rice paddies are a major source of anthropogenic nitrous oxide (N_2_O) emissions, especially under alternate wetting-drying irrigation and high N input. Increasing photosynthate allocation to the grain in rice (*Oryza sativa* L.) has been identified as an effective strategy of genetic and agronomic innovation for yield enhancement; however, its impacts on N_2_O emissions are still unknown. We conducted three independent but complementary experiments (variety, mutant study, and spikelet clipping) to examine the impacts of rice plant photosynthate allocation on paddy N_2_O emissions. The three experiments showed that N_2_O fluxes were significantly and negatively correlated with the ratio of grain yield to total aboveground biomass, known as the harvest index (HI) in agronomy (*P* < *0.01*). Biomass accumulation and N uptake after anthesis were significantly and positively correlated with HI (*P* < *0.05*). Reducing photosynthate allocation to the grain by spikelet clipping significantly increased white root biomass and soil dissolved organic C and reduced plant N uptake, resulting in high soil denitrification potential (*P* < *0.05*). Our findings demonstrate that optimizing photosynthate allocation to the grain can reduce paddy N_2_O emissions through decreasing belowground C input and increasing plant N uptake, suggesting the potential for genetic and agronomic efforts to produce more rice with less N_2_O emissions.

Nitrous oxide (N_2_O) is the third most important greenhouse gas after carbon dioxide (CO_2_) and methane (CH_4_) and is responsible for approximately 6–8% of the current global warming^1^. Additionally, N_2_O enhances atmospheric PM2.5 accumulation and aggravates stratospheric O_3_ depletion^2^,^3^. The concentration of atmospheric N_2_O has increased by 20% from 271 ppb approximately in the year 1750 to 324.2 ppb in 2011 and is predicted to continue to increase^4^. Paddy fields are a major source of anthropogenic N_2_O, accounting for approximately 11% of global agricultural N_2_O emissions^5^. In rice (*Oryza sativa* L.) production areas, the water management practice of alternate wetting and drying and high N input are widely adopted^6^,^7^, which may stimulate N_2_O emissions^8^,^9^. Furthermore, because rice demand is predicted to increase in the coming decades, an expanding rice cropping area and higher N inputs threaten to increase global paddy N_2_O emissions^9^,^10^. Therefore, there is an urgent need to establish sustainable practices for increasing rice yield while reducing N_2_O emissions^11^,^12^. Enhancing plant photosynthate allocation to the rice grain has been widely regarded as an effective strategy of genetic and agronomic innovation for further increases in rice yield^13^,^14^. However, the effects of this strategy on paddy N_2_O emissions are still unknown.

Soil N_2_O is produced through the microbial processes of nitrification and denitrification and through abiotic chemodenitrification reactions^15^,^16^. Among these processes, denitrification is usually considered the dominant N_2_O source^17^. Denitrification is affected by several factors, such as soil carbon (C) availability, soil nitrate availability, soil moisture, soil microbes, and plants^18^,^19^. In particular, plants affect denitrification by providing C as an energy source for denitrifiers through root exudates and/or dead root cells and by mediating soil N availability through N uptake^20^,^21^. This process suggests that the living plants’ photosynthetic products or biomass and their allocation affect N_2_O emissions. For example, van Groenigen *et al*.^21^ showed that enhancing the growth of plants by CO_2_ elevation significantly stimulated N_2_O emissions. Gogoi and Baruah^22^ also found that N_2_O emissions were positively correlated with plant biomass. However, the impacts of crop photosynthate allocation on N_2_O emissions are poorly understood, even though roughly 60% of anthropogenic N_2_O is from agro-ecosystems that determine global food security^1^,^4^.

Greater rice photosynthate partitioning to grain reduces photosynthate allocation to the root and soil^10^,^23^. A decrease in photosynthate allocation to the soil decreases C availability for denitrifying microorganisms, resulting in a reduction in N_2_O production^21^,^24^. Enhanced photosynthate partitioning to the grain could indirectly stimulate total crop photosynthesis, which may increase plant N uptake and, in turn, reduce soil N availability and N_2_O production^25^,^26^,^27^. Therefore, we hypothesized that greater photosynthate allocation to the rice grain reduces paddy field N_2_O emissions, resulting in greater rice yield with less greenhouse gas emissions. We conducted three independent and complementary experiments to test this hypothesis. In experiment 1, we performed a rice variety experiment at the Dangyang (10 varieties) and Jinxian (9 varieties) sites under field conditions to determine the relationships between field N_2_O emissions and the ratio of grain yield to total aboveground biomass, known as the harvest index (HI). In experiment 2, we used a wild-type rice and its mutant with similar biomass of stems and leaves but significant differences in grain yield to examine the effect of photosynthate allocation on N_2_O emissions under field and pot conditions. In experiment 3, we altered rice plant photosynthate allocation by clipping spikelets to explore the underlying mechanisms of photosynthate allocation under field and pot conditions.

## Results

### N_2_O emissions

**Figure 1:**
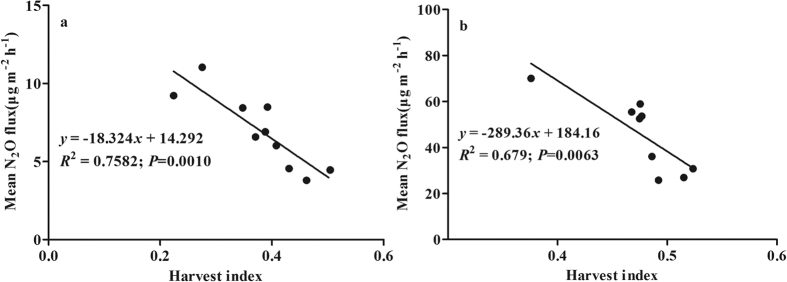
Correlations between mean N_2_O fluxes during the grain filling stage and rice harvest index at Danyang (**a**) and Jinxian (**b**) under the field conditions.

In the rice variety experiment, the mean N_2_O fluxes during the grain filling stage varied significantly among varieties at both sites (Fig. 1, *P* < *0.05*), with up to 142.7% and 171.4% differences between the cultivars with the slowest and fastest N_2_O flux at the Danyang and Jinxian sites, respectively. The mean N_2_O fluxes were significantly and negatively correlated with the HI at both sites (*P* < *0.01*). This correlation indicates that more photosynthate allocation to rice grain (i.e., high HI) reduces paddy N_2_O emissions.

**Figure 2:**
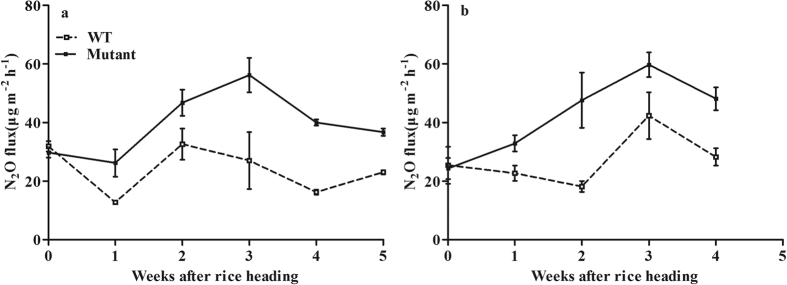
Difference in N_2_O fluxes between the rice variety (WT, Yangdao 6) and its mutant (Mutant) under the field (**a**) and the pot conditions (**b**). Error bars represent 1 standard error.

In the rice mutant experiment, the harvest index values were 0.41 and 0.11 for the wild-type variety (WT) and the mutant (Mutant), respectively. There was no significant difference in N_2_O flux between the WT and the Mutant at the initial heading stage (Fig. 2, *P* > *0.05*). After heading, however, N_2_O fluxes were 84.4% and 69.1% faster in Mutant than in WT under the field and the pot conditions (*P* < *0.01*), respectively.

**Figure 3:**
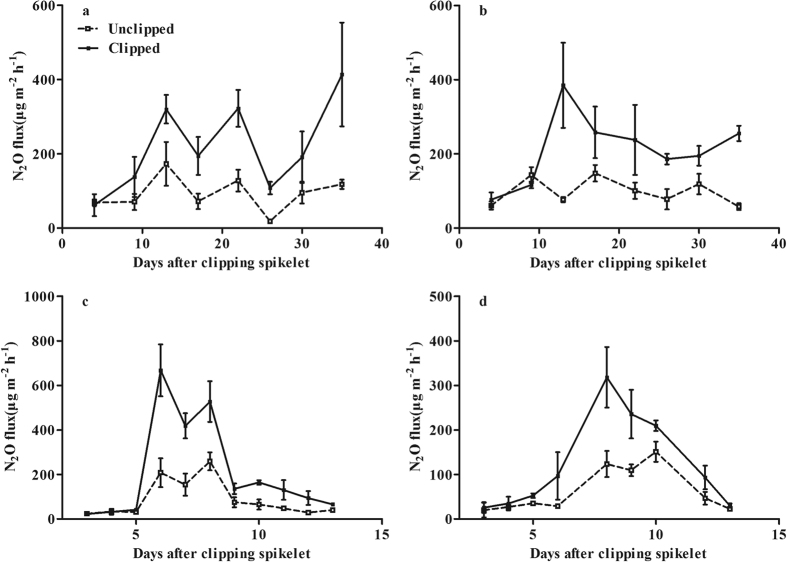
Differences in N_2_O fluxes between the spikelet-clipped and -unclipped treatments under the field (**a**,**b**) and the pot (**c**,**d**) conditions. (**a**,**c**) rice variety Yangdao 6; (**b**,**d**) rice variety Ningjing 1. Error bars represent 1 standard error.

In the spikelet clipping experiment, clipping drastically decreased grain yield and HI (see Supplementary Fig. S1, *P* < *0.01*). However, clipping increased field N_2_O fluxes by 116.9% and 124.6% in Yangdao 6 and Ningjing 1 (*P* < *0.01*), respectively (Fig. 3a,b). Similar results were found in the pot experiment (Fig. 3c,d). In comparison with the unclipped spikelets, clipping significantly stimulated N_2_O fluxes by an average of 154.8% and 67.3% for Yangdao 6 and Ningjing 1 (*P* < *0.01*), respectively.

### Biomass production

**Figure 4:**
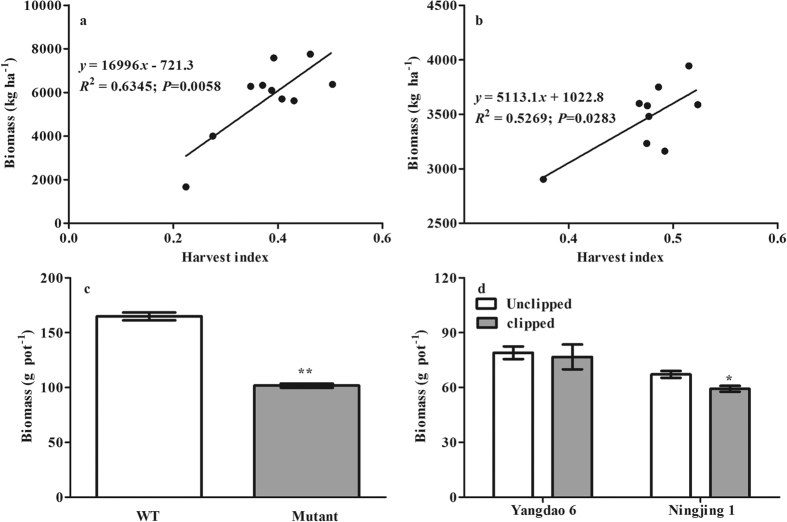
Correlations between biomass accumulation after anthesis and harvest index at Danyang (**a**) and Jinxian (**b**) in the rice variety experiment; differences in biomass between WT and Mutant in the mutant experiment (**c**) and between spikelet-clipped and -unclipped treatments in the spikelet clipping experiment (**d**) under the pot conditions. Error bars represent 1 standard error. *and **indicate significant difference at *P* < *0.05* and *0.01*, respectively.

In the rice variety experiment, the biomass accumulation after anthesis was significantly and positively correlated with the HI at the Danyang (Fig. 4a, *P* < *0.01*) and Jinxian (Fig. 4b, *P* < *0.05*) sites. In the mutant experiment, the biomass of the WT was 62.1% larger than that of the Mutant (Fig. 4c, *P* < *0.01*). In the spikelet clipping experiment, spikelet clipping decreased biomass (Fig. 4d), especially for the Ningjing 1 variety (*P* < *0.05*).

### Plant N uptake

**Figure 5:**
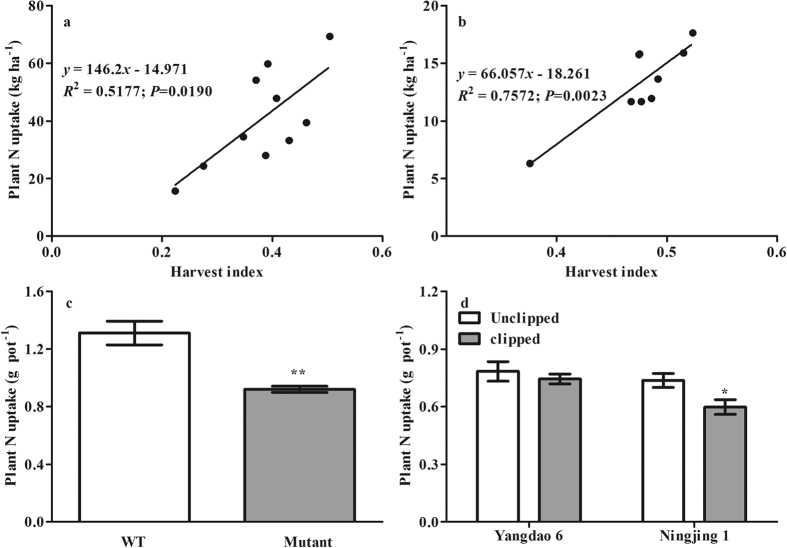
Correlations between plant N uptake after anthesis and harvest index at Danyang (**a**) and Jinxian (**b**) in the rice variety experiment; differences in plant N uptake between WT and Mutant in the mutant experiment (**c**) and between spikelet-clipped and -unclipped treatments in the spikelet clipping experiment (**d**). Error bars represent 1 standard error. *and **indicate significant difference at *P* < *0.05* and *0.01*, respectively.

Plant N uptake after anthesis was significantly and positively correlated with HI at the Danyang (Fig. 5a, *P* < *0.05*) and Jinxian (Fig. 5b, *P* < *0.01*) sites in the rice variety experiment. The plant N uptake of the Mutant was 42.3% lower than that of the WT in the mutant experiment (Fig. 5c, *P* < *0.01*). Spikelet clipping reduced plant N uptake by 5.4% and 23.3% (*P* < *0.05*) in Yangdao 6 and Ningjing 1, respectively, in the spikelet clipping experiment (Fig. 5d). These results indicate that greater photosynthate allocation to rice grain increases plant N uptake, suggesting less soil N availability for N_2_O production.

### Soil dissolved organic carbon and denitrification

**Figure 6:**
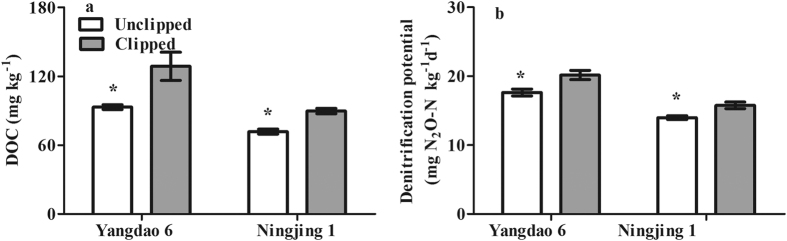
Differences in soil DOC concentration (**a**) and denitrification potential (**b**) between the spikelet-clipped and -unclipped treatments under the pot conditions. Error bars represent 1 standard error. *and **indicate significant difference at *P* < *0.05* and *0.01*, respectively.

In the spikelet clipping experiment, spikelet clipping significantly increased white root biomass (see Supplementary Fig. S2, *P* < *0.05*) and soil DOC concentrations (Fig. 6a, *P* < *0.05*), implying that more photosynthate allocation to grain significantly reduced soil dissolved C. In addition, compared to unclipping, clipping significantly enhanced denitrification potential by 14.3% and 12.8% for Yangdao 6 and Ningjing 1 (Fig. 6b, *P* < *0.05*), respectively, which suggests that more photosynthate allocation to grain significantly reduces soil denitrification for N_2_O production.

## Discussion

**Figure 7:**
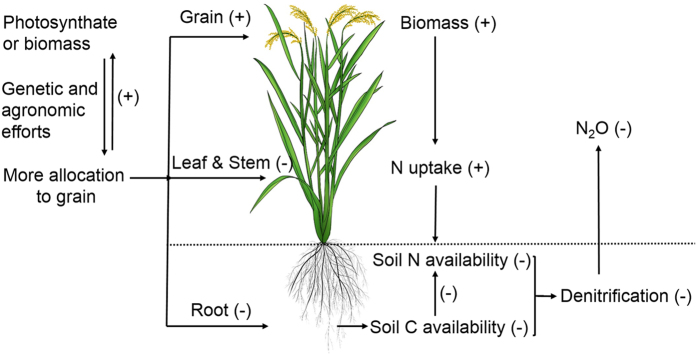
A conceptual framework of the effects of increased photosynthate allocation to grain on N_2_O emissions.

N_2_O emissions varied significantly among rice varieties (Fig. 1), which suggests that varieties may be genetically improved to reduce N_2_O emissions from paddy fields. Gogoi and Baruah^22^ also reported that rice variety could significantly affect soil N_2_O emissions. However, Simmonds *et al*.^28^ showed that there was no significant difference in N_2_O emissions among rice varieties in continuously flooded systems. In the present study, there were significant and negative correlations between N_2_O emissions and HI in the variety experiment (Fig. 1). The rice mutant experiment (Fig. 2) and the spikelet clipping experiment (Fig. 3) further confirmed these correlations by regulating plant photosynthate allocation. Our new findings strongly support the hypothesis that more photosynthate allocation to the rice grain can reduce N_2_O emissions. The underlying mechanisms can be described as enhanced photosynthate allocation to rice grain reducing belowground C input and increasing plant N uptake (Fig. 7).

In the mutant experiment, root exudation and soil DOC content were greater in the Mutant than in the WT (data were published in Jiang *et al*.)^29^, which suggests that more photosynthate allocation to grain can reduce soil C availability for denitrification. Low white root biomass (see Supplementary Fig. S2) and soil DOC content (Fig. 6a) in the unclipped spikelet treatment also suggest that enhanced photosynthate allocation to grain can reduce belowground C input and may then decrease N_2_O production. Previous studies have also shown that spikelet clipping can increase rice root growth and soil C availability^23^,^30^. The spikelet removal technique has been widely used to study the effects of photosynthate allocation on photosynthesis^31^, dry matter accumulation^27^,^32^, N uptake and allocation^27^,^33^, and crop yield^34^. For example, van Der Gon *et al*.^23^ used this technique to demonstrate that a smaller HI can cause high CH_4_ emissions from paddies. Recently, Su *et al*.^35^ further demonstrated that increasing spikelets by adding a single transcription factor gene (barley *SUSIBA2*) could increase rice grain yield and reduce both root growth and CH_4_ emissions. Thus, more photosynthate allocation to rice grain can reduce C allocation to the root and soil, which would reduce C availability for N_2_O production, although we did not measure belowground C allocation in the variety experiment.

Soil N availability is an important regulator of denitrification and is strongly affected by plant N uptake^18^,^24^. The significant and positive correlations between HI and biomass accumulation and N uptake after anthesis (Figs 4 and 5), which were mirrored in the mutant and spikelet clipping experiments, suggest that enhanced photosynthate allocation to the rice grain can greatly increase plant biomass and N uptake. Richards^25^ showed that increasing assimilate allocation to grain induced by large sink size (i.e., spikelet number) should indirectly increase total crop photosynthesis. Mi *et al*.^33^ also found that biomass accumulation and nitrogen uptake after anthesis were regulated by crop sink size. Because Yangdao 6 is a large-panicled variety and the experimental time was short (14 days), clipping spikelets reduced the biomass and N uptake of Yangdao 6 but not significantly (Figs 4d and 5d). Consequently, one likely mechanism underlying the reduction in N_2_O emissions may be soil N limiting denitrification due to the enhancement of rice plant N uptake. In addition, more photosynthate allocation to the soil may increase saprotrophic activity, which would then enhance organic matter decomposition and N mineralization, resulting in an increase in soil N availability for N_2_O production^36^.

Soil O_2_ availability may also play an important role in the reduction of N_2_O emissions caused by optimizing photosynthate allocation. Allocating more C to grain production could indirectly stimulate total crop photosynthesis, which may increase plant transpiration and, in turn, increase soil O_2_ availability^16^,^25^. Increased soil O_2_ availability may decrease N_2_O production, as denitrifying enzymes are expressed under poor oxygen conditions^37^,^38^. In contrast, greater photosynthate allocation to the soil may stimulate heterotrophic metabolism and soil respiration, which, in turn, decreases soil O_2_ availability and creates more suitable denitrifying conditions for N_2_O production^39^,^40^. Chen *et al*.^40^ showed that decomposing organic materials increased O_2_ consumption and anaerobic microsite formation, resulting in enhanced denitrification.

Although increasing photosynthate allocation to grain can benefit rice cropping for improved yield with less N_2_O emissions, the long-term impacts on soil fertility must be taken into account. Shifting more C allocation belowground may modify the composition and activity of the soil microbial community and thus affect the fertility and environment of the soil^39^,^41^. In addition, crop photosynthates are a major source of soil organic matter. A reduction in photosynthate allocation belowground may affect the improvement of soil organic C stocks, which could influence long-term soil fertility^42^,^43^. However, the long-term effects of photosynthate allocation on soil C stocks and fertility are still unknown^43^.

Our findings firstly demonstrate that optimizing rice photosynthate allocation to maximize the HI can reduce N_2_O emissions from paddy fields by decreasing belowground C input and increasing plant N uptake. Recently, we also found that wheat spikelet clipping could increase N_2_O emissions from upland soils (see Supplementary Fig. S3), which implies that greater photosynthate allocation to wheat grain can also depress N_2_O emissions. Crop photosynthate allocation optimization may be implemented by breeding crop varieties with large panicles and refining certain cropping practices, such as proper timing of fertilizer application and good phytosanitary control^13^,^44^. This process is already underway; the HI value for rice has increased from approximately 0.3 to 0.55 since the 1950 s^44^,^45^,^46^. To our knowledge, the maximum global HI value for rice is 0.66^23^, and genetic and agronomic improvements in HI will continue^13^,^14^. The HI can also be affected by climate change^44^, particularly extreme weather events, as well as high temperature and cold stresses. Extreme events can greatly decrease a crop’s HI^47^,^48^, leading to a large reduction in yield and an increase in N_2_O emissions.

## Methods

### Experimental design

#### Experiment 1: Rice variety experiment

The rice variety field experiment was conducted in a rice-wheat rotation cropping system (RW) at Danyang (119.6° E, 32.0° N) and a double rice cropping system (DR) at Jinxian (116.2° E, 28.4° N), two major Chinese rice production areas, in 2011. The climatic conditions, soil properties, and rice varieties are shown in Table S1 (see Supplementary Table S1). Ten and nine varieties released in different eras beginning in 1950 were randomly selected from the RW and DR regions, respectively^49^. Both of these rice varieties are commercial varieties and are commonly planted at over 1,000,000 ha each. The experiment followed a complete randomized block design with three replications. Each plot was 3 m × 4 m in size. Rice seedlings were transplanted on June 30 and April 30 at Danyang and Jinxian, respectively. Nitrogen fertilizer was applied at 225 kg N ha^−1^, of which 50% was used as a base fertilizer before planting, another 30% was used at the tillering stage and the remaining 20% was used at the jointing stage at Danyang. Phosphorus and potassium fertilizers were applied at planting at the rate of 105 kg ha^−1^ and 225 kg ha^−1^, respectively, at Danyang. At Jinxian, nitrogen fertilizer was applied at 150 kg ha^−1^. Nitrogen (50 kg ha^−1^), P (90 kg ha^−1^) and K (180 kg ha^−1^) were applied and incorporated before transplanting. Nitrogen was also applied at tillering (50 kg ha^−1^) and jointing (50 kg ha^−1^) at Jinxian. For all treatments in both experimental sites, water was managed with the same regime by continuous flooding of a 4–5 cm depth of water during the pre-anthesis period and then alternate wetting and drying irrigation during the post-anthesis period. In alternate wetting and drying irrigation, flooding with a 1–2 cm water depth was applied when the soil volumetric water content over a 0–10 cm depth reached approximately 35%. Other management techniques followed local practices at each site.

#### Experiment 2: Rice mutant experiment

To examine the effect of photosynthate allocation on N_2_O emissions, an Indica rice variety (wild-type Yangdao 6, “WT”) and its mutant (Mutant) were tested under field and pot conditions. The grain yield of the WT was significantly higher than that of the Mutant (*P* < *0.01*), whereas no significant differences were observed in the biomass of stems and leaves between the WT and the Mutant^29^. The variety and its mutant were provided by the Institute of Rice, Nanjing Agricultural University.

The field experiment was based on a single rice cropping system and was conducted at Tuqiao experimental station, Nanjing (118.8° E, 32.1° N), China. The soil organic C within the top 15 cm of soil was 13.7 g kg^−1^, and the total N was 1.4 g kg^−1^. There were three replicates in a randomized complete design. Each plot area was 3 m × 4 m in size. Rice seedlings were transplanted manually on June 20, 2012. Nitrogen, phosphorus, and potassium fertilizers were applied at 285 kg ha^−1^, 112 kg ha^−1^, and 171 kg ha^−1^, respectively. Water regimes were similar to those in experiment 1.

The experiment under pot conditions was performed with three replicates at Pailou experimental station, Nanjing. The tested soil was collected from the plow layer of a paddy field at Jiangpu farm, Nanjing. The soil properties were as follows: soil organic C 12.0 g kg^−1^, total N 1.5 g kg^−1^, total P 0.7 g kg^−1^, total K 11.0 g kg^−1^, available N 86.1 mg kg^−1^, available P 22.5 mg kg^−1^, available K 138.3 mg kg^−1^, and pH 6.9 (1:2.5 soil to H_2_O ratio). Plastic pots (diameter, 24 cm; height, 25 cm) were filled with 7 kg soil that was sieved (6 mm mesh size) to remove stones. The seedlings were transplanted into three hills per pot on June18, 2012. To maintain similar plant density and biomass for the WT and Mutant, two plants were planted in the Mutant pots and a single plant per hill was planted in the WT pots (because of the Mutant’s reduced tillering ability)^29^. High rates of N fertilizer were applied according to the local high N input. The basal, tillering and jointing N fertilizers were applied at 165 kg ha^−1^, 99 kg ha^−1^ and 66 kg ha^−1^, respectively. Phosphorus and potassium fertilizers were 88 kg ha^−1^ and 110 kg ha^−1^ applied as basal dressing, respectively. A 2–3 cm water layer over the soil surface was maintained before heading, and then alternate wetting and drying irrigation was applied after heading.

#### Experiment 3: Spikelet clipping experiment

Two rice varieties (an Indica rice, Yangdao 6, and a Japonica rice, Ningjing 1) were tested under field and pot conditions. The field experiment was a randomized complete design with three replicates, which was conducted in 2014 at Jiangpu farm. Each variety had two treatments (i.e., spikelet clipped and unclipped). Each plot area was 3 m × 3 m in size. The soil in experiment 3 was the same as that used in the pot study of experiment 2. The fertilization rate for each plot was 225 kg N ha^−1^, 65 kg P ha^−1^ and 65 kg K ha^−1^. At heading, approximately 50–60% of the spikelets in three plots of each rice variety were removed by clipping them off. The remaining three plots were kept unclipped and served as the control. A water layer of 4–5 cm was maintained during the pre-anthesis period, while alternate wetting and drying irrigation was applied during the post-anthesis period.

A pot experiment with four replicates was performed at Pailou experimental station. Tested soil was collected from the plow layer of the field used for experiment 3. Plastic pots (height, 25 cm; diameter, 24 cm) were filled with 7 kg soil each. To enable more accurate root collection and improve the investigation of white root biomass and root C releases into soils, a nylon root bag (height, 10 cm; diameter, 8 cm; mesh size, 37 μm) was installed in the soil before rice seedling transplanting following the technique described by Lu *et al*.^50^. The root bag was not removed until harvest. Two healthy seedlings were planted in the root bag on June 30, 2014. Nitrogen, phosphorus, and potassium fertilizers were applied as basal dressing at the rate of 165 kg ha^−1^, 88 kg ha^−1^ and 110 kg ha^−1^, respectively. Side-dressing N fertilizer was added at 99 kg ha^−1^, 66 kg ha^−1^, and 33 kg ha^−1^ at the tillering, jointing and booting stage, respectively. Just after the completion of heading, 66% of the rice spikelet was clipped off. The pots with unclipped spikelets were used as the control. A 4–5 cm water layer was maintained before clipping the spikelet. After the spikelet was clipped, water over the soil surface was drained. To maintain normal rice growth, water was added to the root bag after clipping. For Yangdao 6, 200 ml water was added each day from 5 to 14 days after clipping. For Ningjing 1, 400 ml water was added at the 10th day after clipping; thereafter, 100 ml water per day was added. The soil moisture was similar between the clipped and unclipped spikelet treatments. All plants were harvested 14 days after the treatments.

### Measurements

Nitrous oxide fluxes were measured using the static closed chamber technique^29^,^49^. For N_2_O flux measurement in the pot experiment, pots were transferred to a large container with 2 cm water, and a PVC chamber (height, 130 cm; diameter, 30 cm) was placed on the top of each pot. In the field experiment, the chamber (length, 50 cm; width, 50 cm; height, 100 cm) was placed on a PVC frame that was inserted into the soil. Gas sampling was performed once per week between heading and harvest in experiments 1 and 2. In experiment 3, N_2_O fluxes were monitored daily in pots and every 5 days in the field. The N_2_O content was analyzed with a modified gas chromatograph (GC-7890 A; Agilent Technologies, USA). All fresh soils in the rooted compartment were collected and homogenized after harvest in experiment 3. After soil sampling, roots were washed with tap water. White roots were considered new roots because Fe (III) oxides precipitate on rice root surfaces under flooded conditions, causing old rice root to appear brown^51^. Soil dissolved organic C concentrations were measured with a TOC analyzer (multi N/C UV, Analytik Jena AG, Germany). Soil denitrification potential was measured following the technique described by Šimek and Kalčík^52^. Plant samples were oven dried at 105 °C for 30 min and then at 70 °C to achieve a constant weight. Plant nitrogen concentrations were determined using the Kjeldahl method.

### Data analysis

In experiment 1, we analyzed the correlation between the HI and mean N_2_O flux during the grain filling period. In experiment 2, means were tested with the independent-sample t test. In experiment 3, means were analyzed by two-way (i.e., variety and clipping) ANOVA and the independent-sample t test for the given variety. All statistical analyses were performed using SPSS software 11.0. Differences were considered significant at *P* < *0.05*.

## Additional Information

**How to cite this article**: Jiang, Y. *et al*. Optimizing rice plant photosynthate allocation reduces N_2_O emissions from paddy fields. *Sci. Rep.* **6**, 29333; doi: 10.1038/srep29333 (2016).

## Supplementary Material

Supplementary Information
