# Supplementary material for: Optimizing rice plant photosynthate allocation reduces N2O emissions from paddy fields
Source: Sci Rep. 2016 Jul 5;6:29333. doi: 10.1038/srep29333 (PMC4932627; doi:10.1038/srep29333)
Supplement: Supplementary Information [file srep29333-s1.pdf]

Optimizing rice plant photosynthate allocation reduces N_2O emissions from paddy fields

Yu Jiang[1, 2], Xiaomin Huang[1], Xin Zhang[3], Xingyue Zhang[4], Yi Zhang[1], Chengyan Zheng[3], Aixing Deng[3], Jun Zhang [3], Lianhai Wu[5], Shuijin Hu[2], Weijian Zhang[1, 3]*

[1]Institute of Applied Ecology, Nanjing Agricultural University, Nanjing 210095, China;

[2]Department of Plant Pathology, North Carolina State University, Raleigh, NC 27695, USA.

[3]Institute of Crop Sciences, Chinese Academy of Agricultural Sciences / Key Laboratory of Crop Physiology and Ecology, Ministry of Agriculture, Beijing 100081, China

[4]The High School Affiliated to Renmin University of China, Beijing 100080, China

[5]Sustainable Soils and Grassland Systems Department, Rothamsted Research, North Wyke, Okehampton EX20 2SB, UK

*Corresponding author

Weijian Zhang

Institute of Applied Ecology, Nanjing Agricultural University, Nanjing 210095, China

Tel: +86 025 84396030, Fax: +86 025 84396030, E-mail: zwj@njau.edu.cn

Supplementary Table S1 | Climatic conditions, soil properties (0-15 cm), and rice varieties in experiment 1.

Site	Danyang	Jinxian
Mean annual air temperature (°C)	15.0	17.2
Mean annual precipitation (mm)	1058	1549
SOC (g C kg^{-1})	25.0	19.6
Total N (g N kg^{-1})	1.8	1.4
Alkaline hydrolysis N (mg N kg^{-1})	178	95
Rice varieties	Feilaifeng, Tieganqing, Jinnanfeng, Kundao 2, Wufujing, Xudao 2, Yangdao 6, Wuyunjing 7, Ningjing 3, and Zhengdao 11	Nante, Liantangzao, Zhaoyangzao, Xianfeng 1, Zhefu 802, Ganzaoxian 7, Huhongzao 1, Zhongxuan 181, and Jinzao 47

Supplementary Figure S1

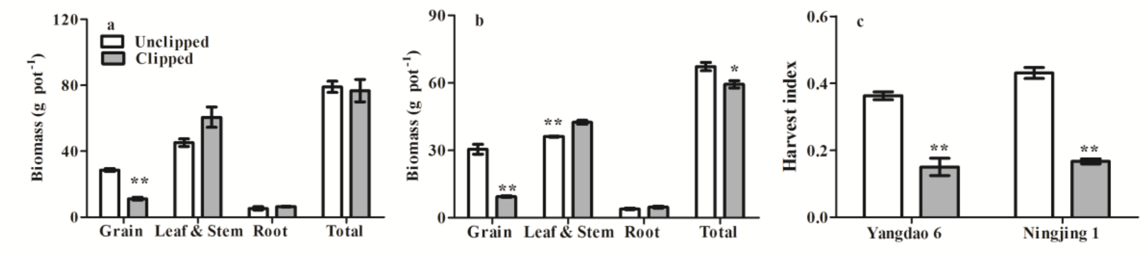

Supplementary Figure S1 | Differences in biomass allocation (a, b) and harvest index (c) between the spikelet-clipped and -unclipped treatments in experiment 3 under the pot conditions. a, rice variety Yangdao 6; b, rice variety Ningjing 1. Error bars represent 1 standard error. * and ** indicate significant difference at *P<0.05* and *0.01*, respectively.

Supplementary Figure S2

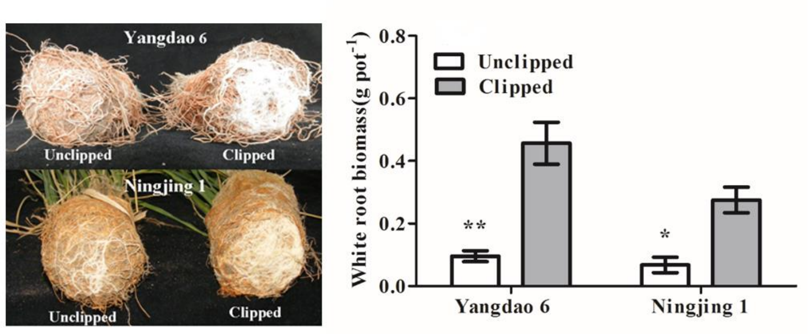

Supplementary Figure S2 | Differences in root growth between spikelet-clipped and -unclipped treatments in experiment 3 under the pot conditions. Error bars represent 1 standard error. * and ** indicate significant difference at *P<0.05* and *0.01*, respectively.

Supplementary Figure S3

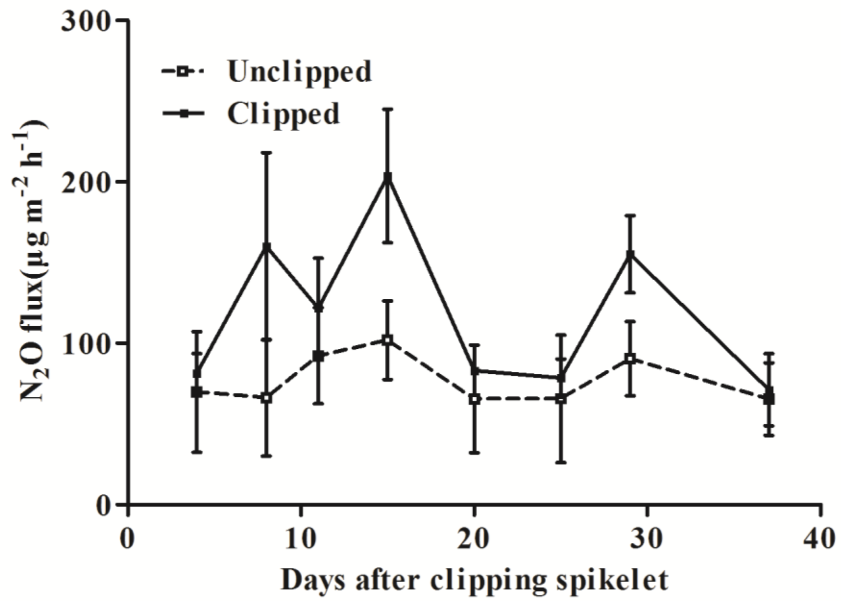

Supplementary Figure S3 | Differences in N_2O fluxes between the wheat spikelet-clipped and -unclipped treatments under the pot condition. Error bars represent 1 standard error.
